# Natural patterns of social support for physical activity participation in newly matched breast cancer survivor dyads

**DOI:** 10.1186/s12905-023-02430-z

**Published:** 2023-05-26

**Authors:** Serena S. Peck, Madison F. Vani, Jenna Smith-Turchyn, Catherine M. Sabiston

**Affiliations:** 1 Institute of Medical Science, Temerty Faculty of Medicine, University of Toronto, Toronto, ON Canada; 2 Faculty of Kinesiology & Physical Education, University of Toronto, 55 Harbord Street, Toronto, ON M5S 2W6 Canada; 3 School of Rehabilitation Science, McMaster University, Hamilton, ON Canada

**Keywords:** Breast cancer, Dyad, Ecological momentary assessment, Physical activity, Social support, Women

## Abstract

**Background:**

Physical activity (PA) can be a beneficial strategy to mitigate physical, emotional, and social-related challenges in women living beyond breast cancer treatment (WBC). However, PA levels among WBC remain low. Optimizing social support provided in a peer-matched setting may increase PA behavior. Unfortunately, factors that lead to an ideal peer-match among WBC are not well understood. The purpose of this study was to contextualize the natural social support environment and PA behavior in newly formed peer WBC dyads participating in an ecological momentary assessment study.

**Methods:**

WBC were matched with a partner and provided with a Fitbit activity tracker. Social support was measured using 21-daily surveys, and a 3-week follow-up survey. Descriptive statistics were calculated. Open-ended survey questions were analyzed using content analysis. Data were analyzed based on (i) social support types (informational, tangible, esteem, and emotional support); and (ii) WBC’ reports of being in a good, neutral, or poor match at the end of the study.

**Results:**

Women (*n* = 46; *M*age = 42.4 ± 7.6 years; 89.2% stage I-III breast cancer) connected with their partner (58.1%) and participated in moderate-vigorous PA (MVPA)(77.1%) on most days over the 21-day study period. Women were identified as being in good (63%), neutral (20%), or poor (17%) dyad matches. The most frequently documented social support received by WBC was esteem support. Participants in a good match were more likely to report receiving all types of social support compared to neutral or poor matches.

**Conclusion & clinical implications:**

Findings describe the social support characteristics important to WBC for facilitating their PA participation in a partner-based setting. This study provides valuable insight that can inform the development of partner-based PA interventions for WBC.

## Introduction

The 5-year survival rate for newly diagnosed patients with breast cancer in Canada is 88% [1]. Women living beyond breast cancer treatment (WBC) are faced with many physical and psychosocial challenges that can last years into survivorship [2–4]. Increasing physical activity (PA) levels are beneficial to the health and quality of life of WBC [5–7]. However, PA levels in most WBC decline after diagnosis [8] and remain low beyond treatment [9]. Social support has been shown to improve PA motivation [10] and participation [11] in individuals living beyond a cancer diagnosis. Yet, there is a lack of social support embedded into current PA programs for WBC, which may impact participation [12].

Drawing on theories of behavior change, such as self-determination theory [13], theory of planned behavior [14], and expectancy-value model [15] social support is a strategy to increase PA behavior through observational learning, shared experiences, increases in intention, and intrinsic motivation [16]. Behavior change PA interventions for WBC can increase PA participation [17, 18], yet researchers have not looked at what specific aspects of social support are perceived by WBC as most useful in facilitating PA behavior. Specifically, social support is the perception or experience that one is cared for and included in a supportive social network [19] and can be categorized into tangible (e.g., products, time), informational (e.g., advice, feedback), emotional (e.g., comfort, motivation), and esteem (e.g., confidence, reassurance) types [20]. Identifying ways to enhance social support in PA programs may be a cost-effective way of enhancing behavioral and health outcomes.

There is evidence that leveraging social support through using a peer-based approach can improve PA [21]. Previous peer-based interventions that had participants matched with a mentor or coach of different disciplines (e.g., exercise professional, trained cancer survivors, or study administrator) improved PA in WBC [22–24]. Additionally, in a peer-matched intervention with a same-sex exercise-trained mentor, those who were in contact with their mentor were more likely to participate in PA [25]. Although interventions using mentors with exercise knowledge may benefit WBC for PA participation, the social support perspective may not be emphasized. Furthermore, the types of social support that may be most conducive to PA are not explored. Matching WBC peers naturally without assigning hierarchical roles (i.e., mentor/mentee) may help us to understand the types of support that is/are valuable for PA among WBC. This information is needed to develop meaningful strategies aimed at improving social support and subsequent motivation for PA participation in WBC during peer-based interventions.

The goal of this daily diary study was to describe the natural social support environment and PA behavior in newly formed peer WBC dyads participating in a daily diary study. The objectives were to: (i) identify characteristics of a positive peer dyadic relationship for PA participation, (ii) describe the natural contextual factors of dyadic social support that WBC found most helpful in facilitating PA participation, and (iii) identify the aspects of social support that WBC perceived as unmet from their matched peer for increasing their PA behavior. The daily diary study design is valuable to capture data from participants early in a dyadic relationship to understand the main drivers of social support in a natural setting.

## Method

This investigation is a secondary analysis of data collected as part of a three-week daily diary study used to examine if dyadic peer-based support could increase PA in WBC. The study was granted appropriate Human Research Ethics Board ethical approval (#00038665). The primary outcomes and detailed methodology are documented elsewhere [26]. In brief, the purpose of the main study was to examine whether various forms of social support received from a matched peer (e.g., information, tangible, emotional, esteem) were associated with increased PA and exercise among WBC. Measured using a Fitbit Inspire HR activity monitor (Fitbit, San Francisco, California), PA is used as a broad definition referring to any bodily movement produced by skeletal muscles and can be characterized as activities of daily life (e.g., occupation, sports, leisure activities such as walking) [27]. Participants were instructed to wear the Fitbit device for all waking hours during the three-week study. Exercise refers to planned, purposeful, structured and repetitive subset of PA with the objective being to improve physical fitness [27]. Exercise was measured using a daily self-report survey. Based on the results of the main study [26] while controlling for baseline exercise levels, higher levels of daily tangible social support for exercise were associated with more daily steps and more light-intensity PA minutes as measured by the Fitbit device. Informational social support was associated with higher moderate to vigorous intensity PA minutes measured by the Fitbit device.

### Participants and procedure

Women living in Canada, diagnosed with primary breast cancer, 18 years of age or older, and regularly participating in less than 150 min of purposeful exercise per week were recruited (*N* = 48). Participants were also required to be cleared for exercise (PAR-Q ± ePARmed-X +), have reliable access to the internet, and agree to wear a Fitbit device every day for three weeks. Exclusion criteria included disclosing any contraindications to regular exercise participation, cancer recurrence, or planned surgery within the study period.

Recruitment for this study spanned June–September 2020 via community cancer organization social media posts (i.e., ActiveMatch, Rethink Breast Cancer, Enliven Cancer Care, and Wellspring). Interested participants emailed the study team and were provided with a consent form. Upon signed consent, an online baseline survey was completed. Using evidence-based criteria of age (within 10 years) and personal characteristics (e.g., geographical location/time zone and cancer severity) [28], the participant was then matched with their peer manually by a research assistant and connected via email. Aligning with a naturalistic study design, women were not instructed on how they should connect (e.g., email, telephone) nor how frequently they should connect with their peer. Participants were mailed a Fitbit device and were instructed to begin the study on a mutually agreed upon start date, within one week of receiving and successfully pairing the Fitbit device. Prior to starting the study, dyads were provided with social support recommendations that were developed for this study (an "exercise partner support guide") and population-level PA guidelines [29]. The “exercise partner support guide” offered suggestions to get to know each other’s exercise story and needs (e.g., ask your partner what they need, share your exercise values, build on what has worked for you in the past), guided partners to help each other set AIMS (Achievable, Important, Measurable, and Specific goals), consider planning what they will do with and for each other (e.g., set an outline for checking in with each other, plan around each other’s schedules, and identify ways to help each other stay on track), and share and celebrate progress (e.g., identify ways to share progress and celebrate each other’s successes, identify rewards that could be shared).

Throughout the study period, participants completed daily end-of-day surveys for 21 consecutive days assessing their perceptions of exercise-related social support. Daily survey links were sent via email at the end of the day (7 pm local time) and a reminder email was sent the following morning (7 am local time) if a response was not received. This process continued for the duration of the three-week study. At the end of the study period (day 22), the dyad was sent a follow-up survey link via email with questions specific to their perceptions of the peer matches and social support. Participants were provided compensation ($5 e-gift card) for every survey completed during the 21-day study period.

### Measures

Participants completed a baseline survey, a daily survey (21 consecutive days), and a follow-up survey (immediately following the 3-week period). They also wore a Fitbit daily for three consecutive weeks during waking hours.

#### Baseline survey

The baseline survey included questions on personal (e.g., age, relationship status, geographical location) and cancer-related (e.g., stage of cancer at diagnosis) information. An additional question asked participants the importance of several reasons for exercising (e.g., increase fitness level, lose weight) on a 4-point scale of 0 = *not at all important* to 3 = *very important*.

#### Daily survey

The daily survey included several closed- and open-ended questions that asked about PA engagement and social support experiences.

##### Exercise participation

To measure exercise participation, women were asked to select whether they exercised that day (1 = *yes*; 0 = *no*).

##### Exercise-related social support

Participants completed several researcher-generated questions. WBC were asked how they connected with their partner that day (i.e., did not connect, in-person, phone, email, text message, virtually [e.g., FaceTime], or other; 1 = *yes;* 0 = *no*). Women were also asked how strongly they agreed with the statement “*All of my support needs around exercise were met today*” on a 5-point scale from 1 = *strongly disagree* to 5 = *strongly agree*. Four items from the Social Support in Sport Survey [30] were used to assess WBCs’ daily exercise-related social support perceptions. For study purposes, the question stem was adapted to: “*Today, my exercise partner supported my exercise by offering…*”. WBC identified the type(s) of support (i.e., tangible, esteem, emotional, and information) that were provided to them by their partner from 1 = *not at all* to 7 = *a lot*. Finally, women were asked two open-ended questions: “*Describe what your exercise partner did or said today to support you around exercise*”, and “*Please share any ideas you have about the type(s) of exercise support that you think would have been helpful to you today*”.

#### Daily PA behavior

A Fitbit assessed PA behavior across 21 days. For this study’s purpose, the number of minutes engaged in PA were described based on Fitbit terminology: lightly active (categorized as light PA), fairly active, and very active (fairly and very active descriptors were summed and categorized as moderate-to-vigorous PA). Individuals living beyond cancer treatments demonstrated good adherence to Fitbits to measure PA and Fitbits demonstrated strong correlations with Actigraph measures [31].

#### Follow-up survey

##### Exercise-related social support

WBC reported how effective it was having an exercise partner to increase PA behavior from 1 = *not at all effective* to 3 = *very effective* and how enjoyable it was having an exercise partner from 1 = *not at all enjoyable* to 3 = *very enjoyable*. WBC were also asked to report if their partner was a good match from 1 = *very poor match* to 5 = *very good match* and to what extent they were similar to their partner from 1 = *not at all similar* to 5 = *extremely similar*. The question on match quality was used to identify profiles of good, neutral, and negative peer matches for this study such that a reporting of a 1 or 2 were labelled poor/negative match, a score of 3 was labelled as neutral, and a score of 4 or 5 was considered a good match. WBC were also asked to select how likely it is that they will continue to communicate with their partner around exercise and how likely it is that they will choose to participate in other partner-based exercise programs with a different partner from 1 = *not at all likely* to 3 = *very likely*. In addition, two open-ended questions on support were asked including, “*Across the past three weeks, what did your partner do to support your exercise that you found helpful?*” and “*Across the past three weeks, what did your partner do to support your exercise that you found unhelpful or not effective?*”.

### Data analysis

Data were screened for outliers and missing data using SPSS (Version 28). Descriptive statistics (frequencies, mean, standard deviation) were computed to provide demographic characteristics of the sample and describe exercise and social support variables. For open-ended questions, a conventional deductive content analysis approach [32] was used, wherein one researcher (SSP) read all open-ended responses to find core themes. Primary coding began with individual participant content analysis guided by the social support dimensions of esteem, emotional, tangible, and informational [20]. Initial codes were based on content explicitly mentioning a specific type of social support. The second round of coding required inference on language and context of responses. Subsequent analysis involved the grouping of responses into positive (i.e., support received) or negative (i.e., support missing) sections. In addition, responses that contained suggestions for how to improve social support among dyads were coded and included within textual descriptions of the groupings. Misplaced or unrelated personal content in responses was coded as ‘not applicable’.

Lastly, the development of good, neutral, and negative match profiles was based on responses to the follow-up survey where participants reported the extent to which they felt their partner was a poor/negative (score of 1 or 2), neutral (score of 3), or good (score of 4 or 5) on a 5-point Likert-type scale for match quality. Descriptive and quantitative data were explored for effect sizes (Hedge’s *g*) of differences in exercise, PA, and social support variables across the match quality groups. The individual narratives (i.e., qualitative data) were then re-examined in their grouping and responses were interpreted on context, tone, and detailed of quotations. Specifically, participants were coded based on match quality (poor/negative, neutral, good) and the open-ended responses were re-read and analyzed using a coding spreadsheet to document codes by match quality profiles. A collective profile description was developed for each group after reading and reviewing the data presented by WBC coded within the three group profiles and were reviewed by two study team members (SSP & MFV), with any disagreements resolved by CMS.

## Results

Data cleaning resulted in the removal of two records. One participant in a dyad dropped out of the study on day four for personal reasons and was therefore removed from the analysis. Since the study was dependent on being in a dyad, the partner of the individual who dropped out was also removed. Participants’ descriptive demographic information for the final analytical sample (*n* = 46) is provided in Table 1. Women were on average 42.4 (range = 28–60) years of age. Most women were diagnosed with stage I-III breast cancer (89.2%), had completed active treatment approximately three years prior to the baseline survey, and 63% were currently receiving hormonal therapy.

**Table 1:** Participant descriptive characteristics (*n* = 46)

Descriptive variables	*M* ± *SD* or *n* (%)
Age, years	42.4 ± 7.6
Education	
High school and/or some university/college	7 (15.2%)
Completed university/college	32 (69.6%)
Started/completed graduate school	7 (15.2%)
Relationship status	
Single	6 (13.0%)
Common law/Married	33 (71.7%)
Separated/Divorced**/**Widowed	6 (13.0%)
Other^a^	1 (2.2%)
Stage of cancer at diagnosis	
0	4 (8.7%)
I	13 (28.3%)
II	16 (34.8%)
III	12 (26.1%)
IV	1 (2.2%)
Number of treatments received	2.5 ± 0.8
Type of treatment received (*n*, % yes)^b^	
Chemotherapy	36 (78.3%)
Radiation	34 (73.9%)
Mastectomy	24 (52.2%)
Lumpectomy	22 (47.8%)
Currently receiving hormonal therapy (*n*, % yes)	29 (63.0%)
Time since active treatment, years^d^	2.9 ± 1.8
Reasons for exercise, *n*(%)^c^	
Prevent recurrence	44 (95.7%)
Prevent disease	42 (91.3%)
Increase strength	40 (87.0%)
Increase fitness	38 (82.6%)
Improve mobility	35 (76.1%)
Reduce stress	34 (73.9%)
Lose weight	27 (58.7%)
Increase social	12 (26.1%)

^a^In a relationship, but not living with partner

^b^Multiple responses given

^c^Numbers represent individuals who endorsed 3 = *very important* for each reason for exercise

^d^*n* = 45

### Descriptive results

**Fig. 1:**
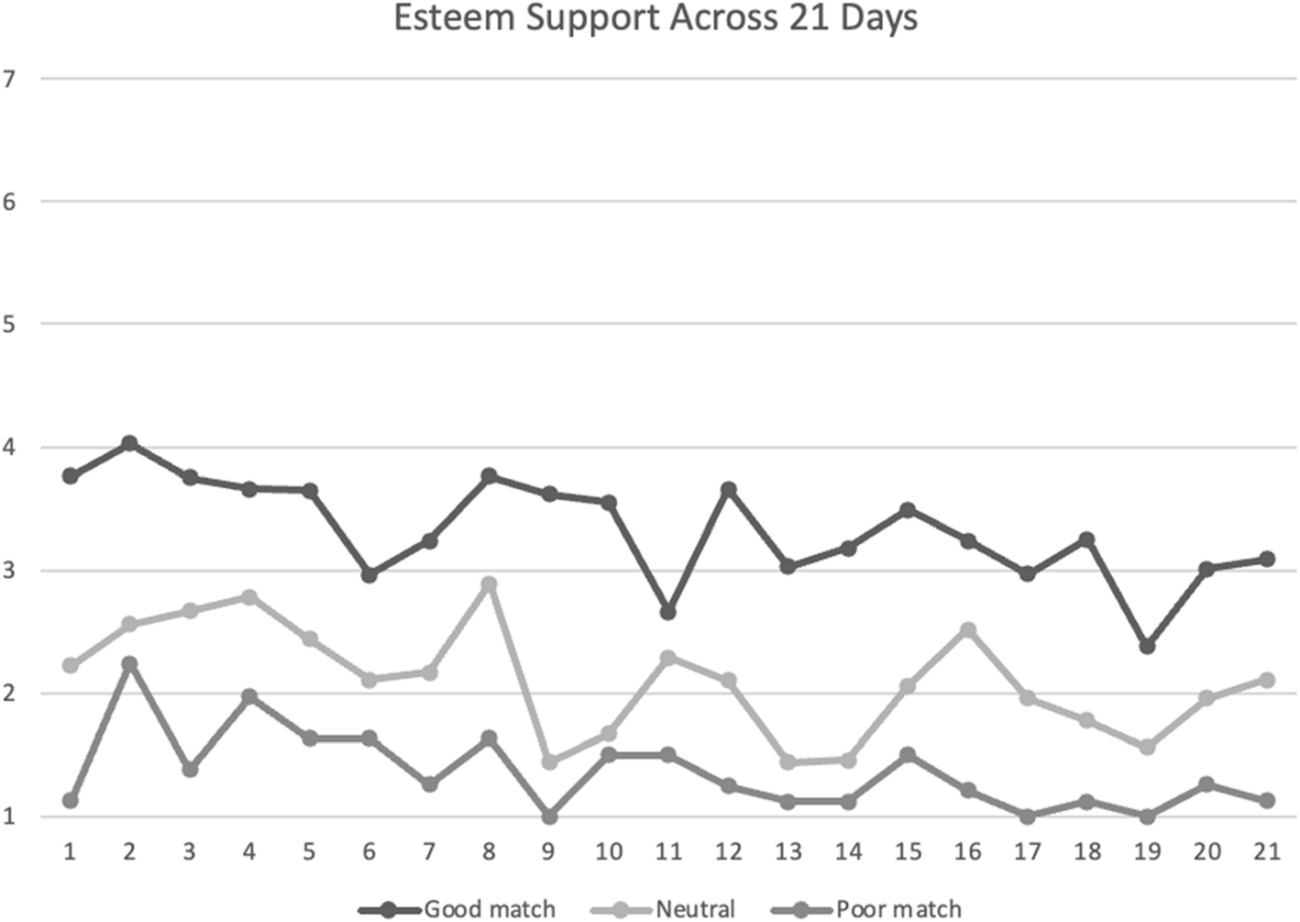
Mean esteem social support scores between participants across 21 days. *Note*. Good match, neutral, poor match are defined by responses to the follow-up survey question: “to what extent was your partner a good match for you.” Social support was assessed on a scale of 1 = *not at all* to 7 = *a lot*

**Fig. 2:**
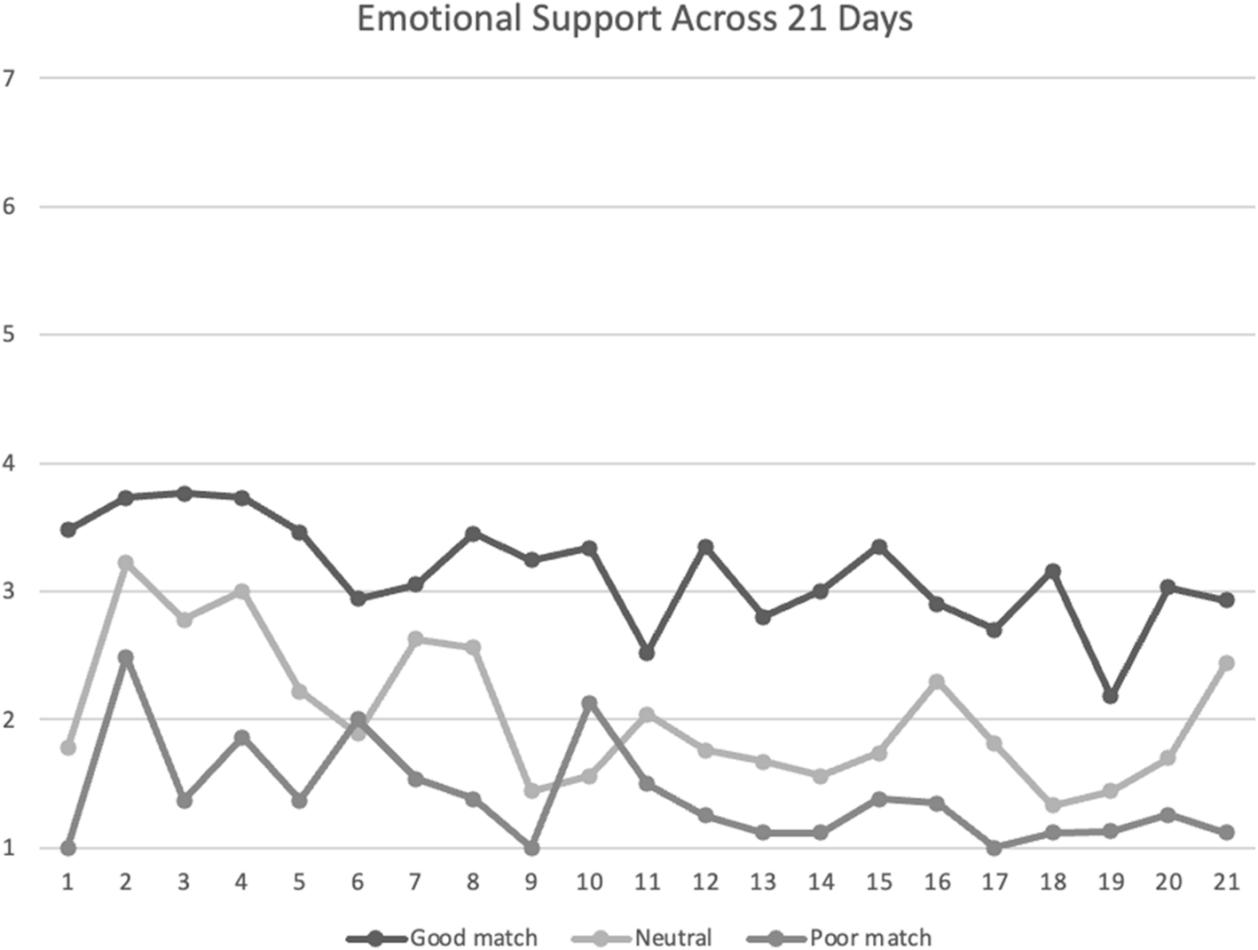
Mean emotional social support scores between participants across 21 days. *Note*. Good match, neutral, poor match are defined by responses to the follow-up survey question: “to what extent was your partner a good match for you.” Social support was assessed on a scale of 1 = *not at all* to 7 = *a lot*

**Fig. 3:**
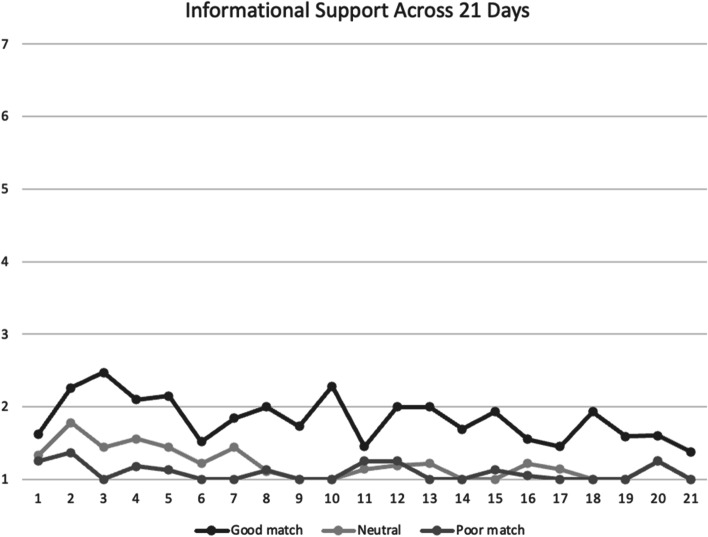
Mean informational social support scores between participants across 21 days. *Note*. Good match, neutral, poor match are defined by responses to the follow-up survey question: “to what extent was your partner a good match for you.” Social support was assessed on a scale of 1 = *not at all* to 7 = *a lot*

**Fig. 4:**
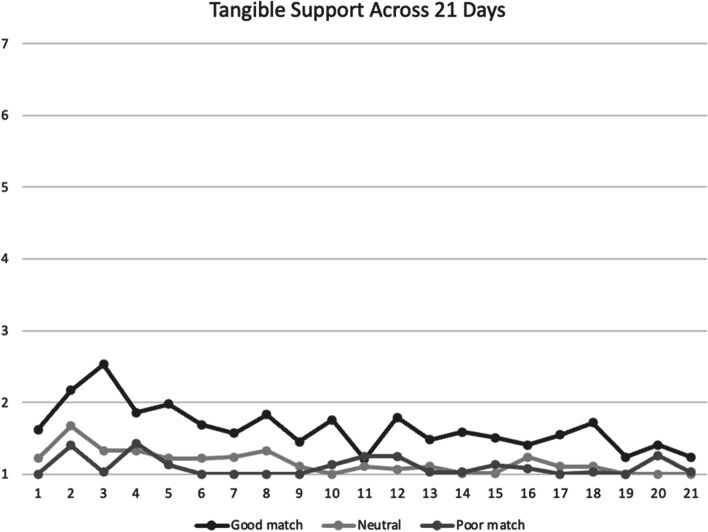
Mean tangible social support scores between participants across 21 days. *Note*. Good match, neutral, poor match are defined by responses to the follow-up survey question: “to what extent was your partner a good match for you.” Social support was assessed on a scale of 1 = *not at all* to 7 = *a lot*

Without providing any guidance on how often participants should connect or exercise, partners connected (58.1%; range = 4–21 days) and exercised (77.1%; range 9–21 days) on over half of the study days. Partners mainly connected using text messages (57.6%) and emails (31.4%) and felt all their support needs were met on 9 of the 21 days. WBC reported the highest scores for esteem (*M* = 2.75) and emotional (*M* = 2.63) exercise support provided by their partners. Mean social support across 21 days for each support type is presented in Figs. 1, 2, 3 and 4. Descriptive information for close-ended daily and follow-up survey responses are presented in Table 2. Results are presented based on the match profiles developed: (a) good (*n* = 29), (b) neutral (*n* = 9), and (c) poor (*n* = 8) match. WBC who identified being in a ‘good match’ reported higher scores on days exercised, days connected, PA, all types of social support, the effectiveness, enjoyment, and similarity of their exercise partner, and higher likelihood of continuing communication with their current partner. Meanwhile, women in a ‘poor match’ had higher scores on the likelihood of future participation with a different exercise partner. The comparisons across groups demonstrated moderate to large effect sizes with the good match group demonstrating much stronger effects of their partnership compared to the poor and neutral match groups.

**Table 2:** Descriptive statistics for physical activity and exercise support variables

**Variable**	**Total** **(*n* = 46)**	**Good match** **(*n* = 29)**	**Neutral** **(*n* = 9)**	**Poor match** **(*n* = 8)**	**Hedge’s** ***g***^**f**^ **Good vs. Neutral/Good vs. Poor/Neutral vs. Poor**
**Daily surveys**					
Days connected with partner, *M*(*SD*)	12.20 (5.00)	13.83 (4.73)	10.78 (4.89)	7.88 (3.00)	.64/1.34/.70
Days exercised, *M*(*SD*)^a^	16.20 (3.28)	17.48 (2.81)	14.00 (3.54)	14.00 (2.14)	1.16/1.29/.00
Did not connect and exercised, *n* days(%)^b^	465 (48.1%)	350 (57.5%)	67 (35.4%)	48 (28.6%)	
Connected and exercised, *n* days(%)	279 (28.9%)	156 (25.6%)	59 (31.2%)	64 (38.1%)	
Connected and did not exercise, *n* days(%)	135 (14.0%)	57 (9.4%)	36 (19.0%)	42 (25.0%)	
Did not connect and did not exercise, *n* days(%)	87 (9.0%)	46 (7.6%)	27 (14.3%)	14 (8.3%)	
Light PA minutes per week, *M*(*SD*)^c^	1661.00 (478.93)	1728.29 (491.04)	1537.60 (314.27)	1555.79 (588.41)	.42/.34/.04
MVPA minutes per week, *M*(*SD*)^c^	255.54 (154.61)	287.75 (175.41)	206.26 (89.10)	194.21 (88.41)	.51/.58/.14
Method of connection, *n*(%)^d^					
Text messages	323 (57.6%)	251 (62.6%)	49 (50.5%)	23 (36.5%)	
Emails	176 (31.4%)	127 (31.7%)	30 (31.0%)	19 (30.2%)	
Phone calls	11 (2.0%)	5 (1.2%)	4 (4.1%)	2 (3.2%)	
Videoconferencing	8 (1.4%)	4 (1.0%)	0 (0%)	4 (6.3%)	
In-person	3 (0.5%)	3 (0.7%)	0 (0%)	0 (0%)	
All exercise support needs met, days, *M*(*SD*)	9.00 (6.29)	10.69 (6.10)	5.89 (6.60)	6.38 (4.87)	.77/.73/.08
Esteem support, *M*(*SD*)^e^	2.75 (1.46)	3.33 (1.42)	2.10 (1.09)	1.36 (0.37)	.91/1.54/.89
Emotional support, *M*(*SD*)^e^	2.63 (1.29)	3.15 (1.27)	2.04 (0.84)	1.40 (0.33)	.93/1.53/.98
Informational support, *M*(*SD*)^e^	1.58 (0.81)	1.83 (0.91)	1.21 (0.36)	1.09 (0.18)	.76/.90/.41
Tangible support, *M*(*SD*)^e^	1.46 (0.77)	1.65 (0.91)	1.16 (0.23)	1.10 (0.15)	.61/.67/.30
**Follow-up survey**					
Exercise partner effectiveness to increase PA, *M*(*SD*)	2.17 (0.74)	2.55 (0.51)	1.56 (0.53)	1.50 (0.76)	1.92/1.85/.09
Enjoyed exercise partner, *M*(*SD*)	2.43 (0.69)	2.79 (0.49)	1.89 (0.33)	1.75 (0.71)	1.96/1.92/.26
Partner similarity, *M*(*SD*)	3.11 (1.29)	3.76 (1.02)	2.44 (0.88)	1.50 (0.54)	1.33/2.39/1.27
Likelihood of future communication with partner, *M*(*SD*)	1.96 (0.70)	2.24 (0.64)	1.78 (0.44)	1.13 (0.35)	.76/1.87/1.62
Likelihood of future participation with another partner, *M*(*SD*)	2.70 (0.59)	2.76 (0.51)	2.33 (0.87)	2.87 (0.35)	.71/.23/.80

*Note*. *PA* physical activity, *MVPA* moderate-to-vigorous physical activity

^a^Self-reported exercise behavior daily for 21 days (i.e., “did you exercise today?”; 0 = no; 1 = yes)

^b^Denominator equals number of participants by number of days (*n* = 21) to yield total observations. For instance, for the total column the denominator would be 966 (46 participants × 21 days = 966 observations)

^c^Objective physical activity behavior measured via FitBit daily for 21 days

^d^Denominator equals instances where participants connected (total days across participants). For instance, for the total column the denominator would be 561 (12.2 days connected × 46 participants = 561 instances)

^e^Average amount of each type of support across the 21 days (scale range = 1–7)

^f^Hedge’s *g* effect size, interpreted as .2 = small, .5 = moderate, and .8 = large effect size

### Content analysis results

Responses to open-ended questions were coded into the four types of social support: (1) informational support, (2) tangible support, (3) esteem support, and (4) emotional support. Within these themes, categories were generated based on commonalities in responses. Informational support included women sharing information (i.e., research articles) with one another. Tangible support included (a) sharing exercise videos, (b) sharing workout plans or challenges, and (c) exercising together (i.e., virtually or in-person). Esteem support included (a) active communication (e.g., updating partner on their workout), (b) creating shared goals (e.g., both partners walk in the evening), (c) exercise encouragement (e.g., sharing motivational texts), and (d) general encouragement or support (e.g., reminding partner to drink water). Emotional support included discussions regarding (a) cancer experiences and (b) exercise experiences.

#### Profile groups

Three profiles of women were created based on their support experience within their dyad and partner similarities. A description of each profile is presented below. Profiles were labeled based on the general pattern of social support experiences described throughout the study: (a) active communication, high support (included 29 women in a ‘good match’); (b) moderate communication, moderate support (included 9 women who were in a ‘neither good nor poor match’, referred to as ‘neutral’ match); and (c) poor communication, low support (included 8 women in a ‘poor match’). The frequency of participant responses in each profile for whether they received or felt they were missing each social support theme and category are shown in Table 3 Across all profiles, esteem support was most frequently documented as received, meanwhile WBC most described tangible and esteem support as lacking. The participants are identified by the dyad pair and partner number (e.g. D1, P1 reflects dyad 1, partner 1).

**Table 3:** Number of women documenting received or missing support for each category over the 21-day period

**Type of Support**	**Category**	**Perceived as met**			**Perceived as missing**		
		**Good match** **(*n* = 29)**	**Neutral** **(*n* = 9)**	**Poor match** **(*n* = 8)**	**Good match** **(*n* = 29)**	**Neutral** **(*n* = 9) **	** Poor match** **(*n* = 8)**
**Informational**	Sharing information	7 (24%)	0 (0%)	0 (0%)	4 (14%)	1 (11%)	0 (0%)
**Tangible**	Sharing exercise videos	5 (17%)	2 (22%)	0 (0%)	1 (3%)	0 (0%)	0 (0%)
	Sharing workout plans or challenges	10 (34%)	1 (11%)	1 (13%)	9 (31%)	4 (44%)	2 (25%)
	Exercising together	2 (7%)	0 (0%)	0 (0%)	12 (41%)	4 (44%)	1 (13%)
**Esteem**	Active communication	24 (83%)	8 (89%)	5 (63%)	9 (31%)	4 (44%)	3 (38%)
	Creating shared goals	12 (41%)	1 (11%)	2 (25%)	1 (3%)	0 (0%)	0 (0%)
	Exercise encouragement	23 (79%)	4 (44%)	1 (13%)	10 (34%)	3 (33%)	1 (13%)
	General support	25 (86%)	1 (11%)	0 (0%)	8 (28%)	4 (44%)	2 (25%)
**Emotional**	Cancer experience	8 (28%)	1 (11%)	3 (38%)	1 (3%)	0 (0%)	1 (13%)
	Exercise experience	2 (7%)	1 (11%)	0 (0%)	1 (3%)	1 (11%)	0 (0%)

##### Active communication, high support

Women in this profile group provided longer and richer descriptions of their partner experience, which were written in a positive tone. For example, when asked to describe the types of support they received, WBC in this profile shared:

She described her exercise plan (length of exercise and steps). We exchanged information about cancer diagnosis. She was enthusiastic about exercising for both of us, she asked about my exercise goals and total steps I wanted to reach for today and if I had reached them. (D17, P1)

I found checking in with my partner to be supportive. We often checked in around goals for the day and [we] gave each other support if we weren't very motivated. Usually checking in late in the day would give that final push to get some more activity in (D5, P1)

Women in this profile also documented greater similarities to their partner (e.g., age, stage in life, exercise goals) and described receiving more support than they felt they were missing. In addition, WBC expressed receiving support across all four support categories. They most frequently reported receiving esteem support, followed by tangible, emotional, and informational support, respectively.

WBC commonly described esteem support as having active communication with their partner. This included their partner texting them in the morning to remind them to exercise, sharing a photo of their walk, or sharing they had completed their daily exercise. Esteem support was also received through exercise encouragement or general support. WBC expressed that their partner sent them motivational texts or positive affirmations and discussed additional topics such as eating habits or engaging in casual conversation. For example, one woman noted, “She’s always very encouraging in her messages ‘You’re doing an amazing job’” (D23, P2) and one woman mentioned “[we] discussed nutrition goals, overall health and motivation” (D8, P2). While WBC commonly reported receiving esteem support, some described instances of wanting to receive *more* esteem support from their partner, in the form of exercise encouragement and active communication. For instance, some WBC desired to have shorter delays between text messages or a message before their walk rather than after: “I didn't hear back from her about the email updating her on my progress” (D1, P1) and “We didn't chat very much today” (D5, P1).

Following esteem support, tangible support was occasionally reported by women in this profile. WBC most often described experiencing tangible support through the sharing of workout plans or fitness challenges to participate in as a pair. This included walking plans, push-up challenges, or exercise pamphlets. Meanwhile, women also documented that even though they were receiving resources from their partner, some of the resources were not their preference (e.g., YouTube vs. outdoor activity): “[I would have liked] different types of exercise suggestions” (D6, P1). Overall, as these women were receiving higher levels of support than others in this study, their comments for what would have been helpful shifted from needs to preferences. For example, one woman expressed their desire to be able to exercise with their matched peer: “While it would be great to have my exercise buddy close by to be able to exercise with, our first conversation last night helped motivate me” (D14, P1).

On occasion, women in this profile described receiving emotional and informational support. Emotional support was provided more often regarding the cancer experience rather than experience with exercise. Women noted it was beneficial to talk about their cancer journey and be able to have someone to empathize with about symptoms or treatment side effects that impacted their PA behavior. Further, informational support was documented as being received through the provision of research articles on cancer survivorship and exercise, resistance training and cancer, and survivorship information from external organizations.

Finally, when women were asked to document the types of support, they thought would have been helpful to receive from their partner, some women’s responses provided the study investigators feedback on what would be beneficial beyond this dyad support. Study suggestions included group exercise, in-person exercise, and being provided workout plans from study staff. For instance, one woman detailed the support received from her partner and exercise-related social support she would like to receive moving forward:

I think even though I did not connect with my exercise partner today, she still challenges me to exercise and provides emotional support from our past conversations. I think it would be helpful to me to have an ongoing exercise group or partner going into the future and longer term to keep the challenge of exercising daily in the front of my mind (D17, P1)

Overall, women in a ‘good match’ shared similar characteristics to their partner, had more active communication over the three weeks, and received support across all support types.

##### Moderate communication, moderate support

Women in a ‘neutral match’ did not respond as often as women in a ‘good match’, gave moderate length and detail in responses, and indicated few similarities with their partner. Women in this profile also listed esteem support as the most frequently received, followed by tangible and emotional support, which were less commonly discussed. The women did not document receiving informational support.

Esteem support in the form of active communication was the most regularly documented, followed by exercise encouragement. WBC articulated that checking in with their partner was useful for accountability and motivational messages encouraged them to exercise: “Just checking in to see if I was active was helpful sometimes, made me feel accountable” (D13, P1). Women also described tangible support received from their partner in the form of exercise videos or challenges was helpful. For instance, one woman described esteem and tangible support received from their partner: “We checked in with each other almost every day, making us accountable to each other. We shared tips, exercises, exercise plan ideas and suggestions and we had a daily planking challenge” (D4, P2). Lastly, only one participant described emotional support in the form of cancer and exercise experiences, wherein they shared conversations about their individual struggles as one way they felt emotionally supported by their peer.

Women mainly expressed wanting more tangible and esteem support. Specifically, WBC stated that their partner provided delayed or minimal communication and described differences in personalities, motivation for the study, and exercise preferences. To illustrate, one woman shared: “She was busy working during the day and wasn’t interested in doing virtual workouts. So, we each did our own thing. Not as motivating for me personally” (D6, P2). Meanwhile, among the women who did receive more communication from their partner, some mentioned the workouts their partner suggested did not interest them, leading them to choose their own workouts. Other women noted that their partner was negative, and this lowered their own motivation to exercise: “[I] receive more complaints than positive messages” (D9, P2). Additionally, due to the perception of a lack of partner support, many women offered suggestions to improve the study with regards to matching participants based on similarities (e.g., schedules), facilitating matched peer introductions and discussions about similar life experiences, and providing opportunities for greater accountability and an exercise partner:

More careful matching of pairs with emphasis on matching of commitments of each person (D9, P2)

Someone to exercise with or to hold accountable for amount of time spent exercising (D13, P1)

Overall, women in a ‘neutral match’ were receiving a moderate amount of support and active communication from their partner, with support received across three categories.

##### Poor communication, low support

Women who were in a ‘poor match’ were much less likely to respond to questions. Responses that were provided were shorter in length with minimal detail (e.g., “Just checked in with me”; D3, P2). WBC also noted that there were few similarities with their partner, and they experienced little to no contact or responses from their partner. For those who did respond, the most frequent support type received was esteem support, followed by tangible and emotional support. There was no mention of informational support.

Women in this profile wanted to receive greater esteem and tangible support. This was mainly attributed to their partner not responding to their calls or texts on a certain day or during the entire study period or indicating they did not want to exercise. And so, due to the lack of communication, WBC documented a higher need for external motivation outside of their partner. For instance, one woman shared that she needed “extra motivation to get out of bed to do it [exercise]” (D16, P2). Meanwhile, another participant described that “touching base with my exercise partner prior to the day starting” (D20, P1) would have helped to improve their exercise motivation. Finally, because of the low communication and support received from their partner, a few women noted wanting advice from study staff on motivation and resources for weight loss or managing cancer side effects and wanting a partner who is equally committed to the study.

## Discussion

Our study aimed to describe the natural social support environment and PA behavior in newly formed peer WBC dyads. The social support women perceived as received or lacking were classified into four categories: informational support, tangible support, esteem support, and emotional support and three distinct participant profiles: high communication, high support (‘good match’), moderate communication, moderate support (‘neutral match’), and low communication, low support (‘poor match’). Women in a ‘good match’ were more similar to their partner (age, marital status), and received more active communication and support across all support categories from their partner. Descriptive findings demonstrated that when no external study influence was provided, WBC matched in peer dyads communicated at least half of the days and exercised over three quarters of the time over a three-week period regardless of being in a good, neutral, or poor match.

The results suggested that across profiles, esteem and tangible support were more readily received, while informational and emotional support were infrequently received from partners. One potential reason for the lack of informational support shared between dyads is because BCW might not have the knowledge to participate in and share exercise concepts (e.g., exercise benefits/advice) [33]. Due to the limited researcher influence in this study, additional support beyond a peer may be required for exercise-related information. The addition of a qualified exercise professional (QEP) or study researcher to provide exercise information to dyads may remove the lack of informational support as a barrier to exercise [34]. As for emotional support, peers were matched without previously meeting, connections were primarily online or virtual, and were not provided with introductions or guidance on how to communicate during the study. This environment may not have facilitated a close enough relationship to provide comfort and understanding regarding exercise experiences. For example, WBC have noted using online support groups for informational support rather than emotional support [35, 36]. Future study designs should incorporate strategies to encourage forming more emotionally support relationships, such as a facilitated dyad introduction to share basic information, previous experiences, and exercise goals.

To better understand how future studies can improve levels of support received from matched peers, describing the reasons for why some BCW felt they were in a better match than others is worthy of discussion. The aspects we identified for an individual to be in a ‘good match’ included (i) having similar characteristics to the partner such as age, marital status, and family responsibilities and (ii) receiving greater active communication from the matched peer, such as increased frequency (once per day). Therefore, considering additional matching criteria may be a way to improve social support in peer dyads. Other studies matching BCW with a peer volunteer have used age, schedule availability, and similar types of cancer treatments for match criteria [22, 37, 38]. Notably, the study that used all three aforementioned peer volunteer matching criteria demonstrated significant improvements in WBCs’ PA over three months [22]. Further, a recent non-exercise specific peer matching preference study with adolescent and young adult living beyond cancer reported that the most preferred matching characteristics were type of cancer, specific support concerns, age at diagnosis, treatment(s) received, and current age [39]. Overall, considering multiple matching characteristics may be an important factor in facilitating a good peer match, and subsequent improvements in social support perceptions and PA behavior.

Women in a successful dyad match also described higher rates of active communication with their partner. Active communication included frequent responses and positive communication in the form of sharing exercise progress and encouragement and motivational messages. These findings are consistent with a randomized control trial of a peer exercise intervention, wherein the use of phone or text support from a peer mentor were described as the “aspects most liked” and “important components” by participants [40]. In the present study, there were no instructions on how often to check-in with each other, nor requirements on sharing PA progress. However, it was apparent that women who were identified as being in a good match showed more initiative to share their exercise progress with their peer. The previously described trial also found that phone calls and text messages from peer mentors can support PA maintenance in WBC [22]. Interestingly, most women in our study connected via text message or email, which may be a barrier to forming a more personal and supportive environment.

Lastly, WBC also noted they wanted more tangible support in the form of workout plans, schedules, and instructions. For women who did receive tangible support from their partner, some noted that the resources provided did not match their preferences. Meanwhile, several women also noted feeling unsure of what exercises to perform or the appropriate duration for exercise engagement. In a study looking at exercise behavior preferences and barriers for WBC, it was reported that needing information from a QEP in addition to social support was important for facilitating exercise participation [41]. Therefore, the addition of written exercise materials and plans or access to a QEP (e.g., physical therapist, occupational therapist, registered kinesiologist), may be beneficial for improving tangible support and facilitating greater exercise participation in WBC dyads.

### Limitations

The results from this observational study should be interpreted with the following limitations. The open-ended descriptions of support experiences ranged in detail and using an alternative method such as interviews could have led women to share additional relevant details. Additionally, the sample size was small and WBC participated in this study in the summer months during the COVID-19 pandemic, where the weather was nicer for outdoor exercise and where exercising provided an option to be outside during COVID-19 restrictions. Additionally, as the COVID-19 pandemic was a socially isolating period, this may have influenced participants willingness to increase their exercise levels and desire for improved social support. Therefore, the results should be interpreted with caution. There is also a lack of generalizability due to the homogeneity of the participant characteristics (e.g., younger age, English speaking, higher education). Overall, further research is required to identify a deeper understanding of the peer support relationship in a more diverse population of WBC.

## Conclusion

In conclusion, matching WBC in peer dyads can improve perceptions of support for exercise participation in WBC. This study highlighted the importance of a good peer match with active communication to provide social support for PA engagement. To improve the dyad relationship, future peer-focused interventions may benefit by matching dyads more systematically, facilitating peer introductions, and providing exercise specific support with QEPs to improve social support for PA participation.

## Data Availability

The data generated during the current study are not publicly available but are available from the corresponding author on reasonable request.

## Abbreviations

PA: Physical activity
QEP: Qualified exercise professional
WBC: Women living beyond breast cancer treatment
